# Thrombolome and Its Emerging Role in Chronic Kidney Diseases

**DOI:** 10.3390/toxins13030223

**Published:** 2021-03-18

**Authors:** Justyna Fryc, Beata Naumnik

**Affiliations:** 1st Department of Nephrology and Transplantation with Dialysis Unit, Medical University of Bialystok, Zurawia 14 St., 15-450 Bialystok, Poland; bnaumnik@poczta.onet.pl

**Keywords:** thrombolome, thrombosis, uremic toxins, chronic kidney disease, indoxyl sulfate, indole-3-acetic acid, kynurenine, p-cresol sulfate, p-cresol glucuronide, phenylacetylglutamine, trimethylamine N-oxide

## Abstract

Patients with chronic kidney disease (CKD) are at an increased risk of thromboembolic complications, including myocardial infarction, stroke, deep vein thrombosis, and pulmonary embolism. These complications lead to increased mortality. Evidence points to the key role of CKD-associated dysbiosis and its effect via the generation of gut microbial metabolites in inducing the prothrombotic phenotype. This phenomenon is known as thrombolome, a panel of intestinal bacteria-derived uremic toxins that enhance thrombosis via increased tissue factor expression, platelet hyperactivity, microparticles release, and endothelial dysfunction. This review discusses the role of uremic toxins derived from gut-microbiota metabolism of dietary tryptophan (indoxyl sulfate (IS), indole-3-acetic acid (IAA), kynurenine (KYN)), phenylalanine/tyrosine (p-cresol sulfate (PCS), p-cresol glucuronide (PCG), phenylacetylglutamine (PAGln)) and choline/phosphatidylcholine (trimethylamine N-oxide (TMAO)) in spontaneously induced thrombosis. The increase in the generation of gut microbial uremic toxins, the activation of aryl hydrocarbon (AhRs) and platelet adrenergic (ARs) receptors, and the nuclear factor kappa B (NF-κB) signaling pathway can serve as potential targets during the prevention of thromboembolic events. They can also help create a new therapeutic approach in the CKD population.

## 1. Introduction

Despite the advances in the prevention and treatment of thromboembolic complications, patients with chronic kidney disease (CKD) are at an increased risk of a spontaneously induced thrombosis, paradoxically associated with bleeding complications [1,2]. The causes of hypercoagulability and bleeding disorders seen in patients with CKD are multifactorial and not fully understood despite many experimental and clinical studies conducted in this field [3]. Traditional risk factors for thrombotic events do not fully explain an increased tendency to develop major cardiovascular events such as myocardial infarction (MI), strokes, or deep vein thrombosis (DVT) complicated by pulmonary embolism in the renal insufficiency population. Therefore, cardiovascular disease (CVD) remains a significant cause of mortality and morbidity among the CKD patients worldwide [4,5].

A growing body of evidence points to an important role of non-classical risk factors in inducing a prothrombotic phenotype characterized by an increased risk of both arterial and venous thrombosis in the population with renal impairment. Recently, attention has been paid to the significant role of uremic toxins, as new risk factors for these complications. A unique CKD-specific risk factor is a group of uremic toxins generated in the gut, which potentially connect CKD and the occurrence of CVD [6]. Gut microbiota-derived uremic toxins linked in human and animal studies to thromboembolic complications associated with renal impairment mainly include metabolites of dietary tryptophan (TRP) (indoxyl sulfate (IS), indole-3-acetic acid (IAA) and kynurenine (KYN)), phenylalanine/tyrosine (p-cresol sulfate (PCS), p-cresol glucuronide (PCG), phenylacetylglutamine (PAGln)), and choline/phosphatidylcholine (trimethylamine N-oxide (TMAO)). This group of inflicting thrombosis uremic solutes, generated by gut microbiota and retained with renal impairment, is named a “thrombolome”, which merges the words “thrombosis” and “metabolome” [7]. As the gut–kidney–vascular axis has been recently highly researched as a factor contributing to the development of thrombotic events among CKD patients, this review summarizes the most recent developments in the field of gut microbiota-generated uremic toxins and their impact on thrombosis mechanisms in renal insufficiency.

## 2. Hemostatic Disorders in CKD

Uremic patients are more prone to thrombotic complications and associated hemostatic disorders. Clinically, it can manifest as acute thrombotic occlusion of arteries causing MI or stroke, spontaneous venous thrombosis resulting in pulmonary embolism, hemodialysis access dysfunction, as well as gastrointestinal tract or intracranial bleeding [8,9,10,11]. Compared to controls with normal renal functions, the risk of venous thrombotic events is 2.5-fold higher during stage 3 and 5.5-fold higher during stages 4–5 of CKD [12,13]. The pulmonary embolism incidence increases from 66/100,000 in people with normal renal function to 204/100,000 in CKD and 527/100,000 in end-stage renal disease (ESRD) patients [14].

Hypercoagulability is associated with increased plasma levels of procoagulant factors, the production of procoagulant microparticles, and endothelial dysfunction. Higher levels of factor VII, factor VIII, PAI-1, thrombin-antithrombin complex, and tissue factor (TF) were found in patients with uremia [15,16,17]. Additionally, CKD influences the dynamic process of thrombus formation by alterations in components such as cellular adhesion molecules (CAMs) expression and disturbances in the extracellular matrix of the vessel wall. Accelerated atherosclerosis in CKD translates into an increased risk of thromboembolic events development [18]. It is mainly caused by the formation of a thrombus on a ruptured atherosclerotic plaque in the artery wall. The atherothrombotic process is triggered by platelet adhesion and aggregation on the exposed vascular surface and the activation of the clotting cascade.

On the other hand, the reasons for a greater risk of venous thromboembolism (VTE) among CKD patients are not well understood. Despite numerous studies confirming the association between CKD and VTE, the mechanisms and role of uremic toxins in this phenomenon are poorly described [13,19,20,21,22,23]. The increased risk of VTE may be partially explained by the activation of procoagulants, decreased endogenous anticoagulants, disturbed platelet activation and aggregation, decreased fibrinolytic activity, and inflammatory state in kidney failure [24,25]. The Multiple Environmental and Genetic Assessment of risk factors for venous thrombosis (MEGA) study, as well as the Reasons for Geographic and Racial Differences in Stroke (REGARDS) study, proved that increased levels of factor VIII and von Willebrand factor (vWF) are responsible for the association between CKD and VTE [26,27].

A key factor in generating an occlusive thrombus is increased platelet activation and aggregation. On the other hand, the inadequate function of platelets, typically found in uremia, is also a cause of excessive bleeding. Furthermore, the bleeding risk is additionally triggered by abnormalities in coagulation cascade factors and an intensified activity of the fibrinolysis [2]. Disturbances of platelet function in uremic patients are caused by the defective arachidonic acid metabolism, cyclooxygenase (COX) activity, phospholipase A2 activity, and a decrease in the production of thromboxane A2 (TXA2) [28,29]. Furthermore, hemostatic disorders are triggered by inflammation, oxidative stress, fluid retention, and anemia in patients with renal insufficiency [30,31].

CKD is a risk factor for both spontaneous venous and arterial thrombosis. Mechanisms responsible for the development of thrombus in the arterial and venous bed are different. The damage of endothelial cells and exposure of underlying subendothelial matrix and vascular smooth muscle cells (vSMCs) increases the risk of arterial thrombus formation [32]. On the other hand, venous thrombosis can happen spontaneously on an intact endothelial layer mainly due to endothelial microparticle release. Therefore, anti-platelet agents are recommended in arterial thrombosis, while drugs influencing the coagulation cascade should be used in venous thrombosis. However, the use of antiplatelet agents must be carefully considered because of their ability to increase the bleeding risk.

## 3. Dysbiosis in CKD

Intestinal bacteria facilitate many beneficial physiological processes and generate some active metabolites. The microbiota in the human gastrointestinal (GI) tract exceeds 3.8 × 10^13^ microorganisms and weighs about 200 g [33]. It has the potential to produce thousands of metabolites due to a vast and diverse array of microbial cells. The microbiome encodes over three million genes, whereas the human genome consists of about 23,000 genes [34]. Therefore, the gut microbiota is considered as the body’s largest endocrine organ. However, many diseases and their treatment can disrupt the composition of intestinal flora. The association between alterations of fecal microbial community and CKD was first discovered in the 1970s [35]. As a result of a disruption in microbial composition and stability, known as dysbiosis, there is a potential to generate harmful metabolites and develop a wide range of diseases such as obesity or CVD [36]. Also, it has been shown that gut dysbiosis can trigger a higher risk of thromboembolic complications found in patients with CKD [37]. It is well known that gut microbiota impacts the production of hepatic blood coagulation proteins and platelet function by affecting the synthesis of vitamin K and hepatic vWF as well as regulating serotonin biosynthesis [38,39,40]. Besides the influence on the afore-mentioned components, gut microbiota can have a negative impact on the functioning of the coagulation system by producing uremic metabolites and solutes. Therefore, the GI tract-kidney axis can modulate thrombotic potential in patients with CKD.

In CKD, due to the decrease in the glomerular filtration rate (GFR) and impaired tubular secretion, there is an increase in the level of nitrogen compounds such as urea and uric acid. Instead of being excreted via the urinary tract, they are partially eliminated through the intestines. In the gastrointestinal system, urea is converted to ammonium, which causes pH elevation and mucosal damage [41]. This can further exacerbate gut dysbiosis. Also, certain external factors, including a CKD-specific diet or gut edema in patients with protein loss, impact the composition of intestinal microflora (Table 1) [42]. Additionally, antibiotics, oral phosphate-binding agents, and iron supplements (frequently used by CKD patients) influence the gut microbiota environment. Finally, the uremic milieu and impaired protein assimilation cause an influx of undigested proteins into the distal intestine that favors the overgrowth of proteolytic bacteria such as Enterobacteriaceae and *Escherichia coli*, which metabolize nitrogen compounds and further increase the production of harmful uremic toxins [43]. This process decreases the prevalence of saccharolytic genera such as Bifidobacteria, Lactobacilli, Eubacteria, Bacteroides, and Prevotella, which are the “good” microbiota involved in the production of short-chain fatty acids (SCFA) from dietary fiber. The SCFAs play a crucial role in protecting the cardiovascular system [44].

## 4. Gut Microbiota-Derived Uremic Toxins

Disturbances in the intestinal microbial community in CKD are associated with an increased production of gut-derived uremic toxins such as IS, IAA, KYN, PCS, PCG, PAGln, and TMAO. The accumulation of uremic toxins in renal failure is highly thrombogenic and exerts toxic effects on the vessel wall [45]. This is possibly due to the induction of platelet dysfunction and modulation of their responsiveness as well as harmful effects on vascular endothelium and a decreased production of nitric oxide [46]. It was determined that TMAO can directly modulate platelet hyperresponsiveness and increase the clot formation rate. An increased level of IS can inhibit endothelial proliferation and enhance the production of highly thrombogenic endothelial microparticles.

Uremic solutes are retained in blood and tissues because their excretion is limited due to kidney function impairment. They are classified by their protein binding properties and ability to remove from the blood during dialysis. PAGln and TMAO are not protein-bound uremic toxins and they are cleared by the kidney through tubular secretion and easily removed by dialysis in the case of ESRD. On the other hand, IS and PCS are protein-bound solutes, also cleared by the kidney through tubular secretion, however, they are poorly removed during dialysis in the case of kidney failure.

## 5. Tryptophan-Derived Uremic Toxins

### 5.1. Indoxyl Sulfate and Indole-3-Acetic Acid

Indoxyl sulfate (IS) and indole-3-acetic acid (IAA) are pro-thrombotic small protein-bound uremic toxins, that belong to the panel of thrombolome [47,48]. They derive from the dietary TRP metabolization by intestinal bacterial tryptophanases, employing the indolic pathway. Indole is produced in the colon and absorbed into the blood circulation through this pathway. This process leads to the biosynthesis of IS in the liver. IAA is directly produced in the intestines from TRP metabolism, or endogenously in tissue via tryptamine. IS and IAA are excreted in urine through tubular secretion mediated by organic anion transporters OAT1 and OAT3 [49].

The serum levels of IS and IAA significantly increase during the progression of CKD. Compared to healthy people, the IS concentration can increase more than 50 times as GFR decreases [50,51,52]. Due to the fact that IS and IAA are protein-bound, poorly dialyzable compounds, there are no available methods for their effective removal in ESRD. The reduction rate of blood IS concentration after hemodialysis is only about 30% [53].

Indolic uremic compounds (IS and IAA) have a multi-directional impact on the body. They influence almost all the components in the pathogenesis of thrombosis and hemostasis. The prothrombotic properties of IS and IAA have been proven both in vitro and in animal studies [54,55]. The evidence that these molecules are partially responsible for thrombotic events in CKD patients were also presented in certain clinical cohorts (473 participants with advanced CKD from the Dialysis Access Consortium Clopidogrel Prevention of Early AV Fistula Thrombosis trial and 377 participants (many of whom had CKD stage 2–3) from the Thrombolysis in Myocardial Infarction II trial) [56]. A higher level of IS was associated with an increased risk of dialysis vascular access thrombosis (especially after endovascular interventions) and positively correlated with the number of thrombectomy procedures in the hemodialysis patients [57,58].

The probable procoagulant mechanisms induced by IS and IAA are related to increased levels of tissue factor/factor VII complexes, platelet activation, and harmful effects on the endothelium [55,56]. Additionally, IS promotes thrombosis by its ability to enhance the procoagulant activity of red blood cells (RBCs) through phosphatidylserine exposure and increases endothelial microparticle (EMP) release [59,60]. Also, IS can trigger RBC shrinkage and RBC cell membrane scrambling that lead to suicidal erythrocyte death, which can impede microcirculation [61].

#### 5.1.1. Platelet Hyperactivity

Indolic compounds contribute to CKD-associated thrombosis by IS-induced platelet hyperactivity, including elevated response to collagen and thrombin, an increase in platelet-derived microparticles, and platelet-monocyte aggregation. The mechanisms responsible for platelet adhesion and aggregation are related to and enhanced by IS P-selectin and GPIIb/IIIa expression in the presence of a low concentration of collagen and thrombin [62]. Additionally, one of the key regulators for platelet activation includes oxidative stress [63]. IS has a distinct ability to induce the production of reactive oxygen species (ROS) and it was proved that platelet activity can be enhanced through the activation of ROS-mediated p38 mitogen-activated protein kinase (p38 MAPK) signaling pathway [62,64].

#### 5.1.2. Endothelial Cells

IS is deleterious for the endothelium [65]. It inhibits the proliferation and viability of endothelial cells, reduces the production of nitric oxide, and impairs endothelial progenitor cells [66,67]. Furthermore, IS induces free radical generation, oxidative stress, and progressive inflammatory processes that have harmful effects on vascular endothelium and play a potential role in the progression of vascular and hemostatic dysfunctions [68,69]. Additionally, IS stimulates endothelium for the secretion of chemokines as well as cytokine-induced, hemostasis-related molecules [70,71]. In cultured human endothelial cells, IAA also induced endothelial inflammation, oxidative stress, activation of an aryl hydrocarbon receptor (AhR), and the inflammatory non-genomic AhR/p38MAPK/NF-κB pathway that induced the pro-inflammatory enzyme cyclooxygenase-2 [72].

#### 5.1.3. Tissue Factor

Indolic uremic toxins increase TF protein expression in ECs, vSMCs, and peripheral blood mononuclear cells (PBMCs) [73]. They upregulate vSMC TF levels by increasing TF stability and decreasing its ubiquitination [32]. TF is the initiator of the extrinsic coagulation pathway and a procoagulant protein enhancing thrombosis [74,75]. Usually, under normal conditions, TF is not expressed by ECs. Its expression in ECs is mediated by pro-inflammatory cytokines, lipopolysaccharides (LPS), growth factors, interleukin-1 beta (IL-1β), tumor necrosis factor-alpha (TNF-α), thrombin, and vascular endothelial growth factor (VEGF). These factors act by various intracellular signaling pathways and via different transcription factors, including NF-κB, activating protein 1 (AP-1), nuclear factor of activated T-cells (NFAT), and early growth response protein 1 (Egr-1) [74,76].

**Figure 1:**
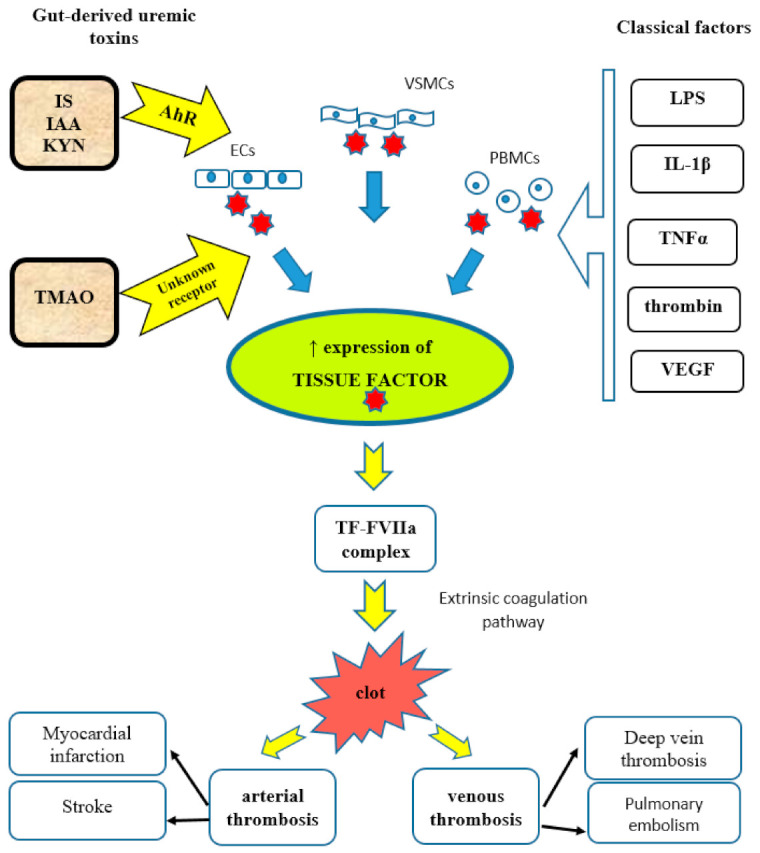
Mechanisms of increased tissue factor (TF) expression and TF-induced thrombosis. IS, indoxyl sulfate; IAA, indole-3-acetic acid; KYN, kynurenine; TMAO, trimethylamine N-oxide; AhR, aryl hydrocarbon receptor; ECs, endothelial cells; VSMCs, vascular smooth muscle cells; PBMCs, peripheral blood mononuclear cells; LPS, lipopolysaccharide; IL-1β, interleukin-1 beta; TNFα, tumor necrosis factor alpha; VEGF, vascular endothelial growth factor; TF-FVIIa complex, tissue factor-factor VIIa complex.

The induction of TF by indolic toxins occurs via the activation of the AhR [73]. IS levels correlate significantly with TF activity and vSMC AhR activity [77]. Recently, it was discovered that AhR regulates TF through STIP1 homology and U-Box containing protein 1 (STUB1) [78]. Endothelial expression and procoagulant activity of TF, induced by IS and IAA, is mediated by the AhR non-genomic inflammatory pathway that involves p38 MAPK, NF-κB, and AP1 translocation [79]. Activation of AhR leads to increased levels of TF/factor VII complex and promotes arterial thrombosis [56] (Figure 1). Therefore, AhR antagonists can serve as a novel class of anticoagulants in the case of kidney insufficiency. It was also proved that targeting the STUB1–TF axis normalizes hyper-thrombotic uremic phenotype without increasing the bleeding risk [78].

### 5.2. Kynureine Metabolites

The thrombolome panel contains uremic toxins derived also from the kynurenine (KYN) pathway. It includes metabolites generated via increased degradation of TRP to KYN such as 3-hydroxyanthranilic acid (3-HAA), 3-hydroxykynurenine (3-HKYN), kynurenic acid (KYNA), anthranilic acid (AA), and quinolinic acid (QA). The KYN cascade, mediated by TRP 2,3-dioxygenase (TDO) and indoleamine 2,3-dioxygenase (IDO) enzymes, is the main metabolic route leading to the degradation of 95% of dietary TRP.

Altered kynurenine metabolism and increased serum KYN/TRP ratio, as a result of an increased TRP breakdown, are detected in patients with CVD such as coronary heart disease or stroke [80,81,82,83]. Also, in ESRD patients TRP breakdown to KYN metabolites caused a decrease in TRP level which is reflected by the increased KYN/TRP ratio [84,85]. The KYN pathway is linked with hypercoagulability and CVD among the CKD population [86,87]. In maintenance hemodialyzed patients, the KYN/TRP ratio is positively associated with thrombosis markers such as thrombomodulin and von Willebrand factor [88]. Additionally, KYN metabolites were reported to be significantly associated with elevated prothrombin factors 1 + 2 in dialysis patients [87].

KYN pathway-derived uremic toxins enhance thrombosis mainly through activation of AhR [56,89]. Summarizing, the KYN pathway dysregulation contributes to thrombus formation by TF overexpression, deregulation of plasma coagulation factors, induction of endothelial cell dysfunction, increased oxidative stress and inflammation, as well as the progression of atherosclerosis [87,88,90,91,92,93].

## 6. Phenylalanine/Tyrosine-Derived Uremic Toxins

### 6.1. P-Cresol Sulfate and P-Cresol Glucuronide

P-cresol sulfate (PCS) and p-cresol glucuronide (PCG) are the circulating metabolites of bacterial fermentation from phenylalanine and tyrosine. PCS is a protein-bound uremic toxin that derives from endogenous sulfate conjugation of p-cresol in the liver, and PCG derives from glucuronide conjugation in the enterocytes [94]. Both of them are excreted in urine through tubular secretion. However, with progressing renal failure, p-cresol metabolites accumulate with a shift from sulfation to glucuronidation, and therefore, the PCS to PCG ratio decreases during CKD progression [95]. Additionally, because PCS and PCG are protein-bound particles, they are poorly cleared with dialysis. PCS levels are around 200-fold higher than PCG [96]. A higher total p-cresol metabolites level (PCS + PCG) and a lower PCS to PCG ratio are independently associated with mortality [96]. P-cresol sulfate directly contributes to endothelial dysfunction influencing endothelial microparticles (EMPs) release and is associated with CVD in HD patients [97,98,99].

#### Endothelial Microparticles

Microparticles are small membrane vesicles originating from platelets, leukocytes, as well as ECs and act as prothrombotic stimulants and proinflammatory mediators [2,100]. Their level is increased in patients with acute coronary syndrome, ischemic stroke, severe hypertension with end-organ damage, and venous thromboembolism, where they indicate an endothelial injury and are linked to thrombosis [101]. It was proven that the level of EMPs starts to increase during the progression of CKD [100,102,103]. P-cresol metabolites are prominent among uremic toxins that stimulate the release of EMPs [104,105].

### 6.2. Phenylacetylglutamine

The phenylacetylglutamine (PAGln) is a gut microbiota-derived metabolite of essential amino acid phenylalanine (Phe). Phenylalanine is mainly absorbed in the small intestine, but unabsorbed Phe reaches large intestines and is metabolized by gut bacteria. The microbial porA gene facilitates the conversion of dietary phenylalanine into phenyl pyruvic acid and subsequently into PAA (phenylacetic acid) [106]. PAA enters the portal system and is metabolized in the liver to generate PAGln and phenyl acetyl glycine (PAGly), which both promote platelet responsiveness and thrombosis potential [107]. PAGln and PAGly levels were shown to be significantly increased in subjects with renal failure [108]. There is a relationship between PAGln level and thrombotic events in humans primarily based on platelet adrenergic signaling modulation [107]. PAGln is associated with major cardiovascular events such as MI, stroke, and death [108].

#### Platelet Thrombosis via Adrenergic Receptor Signaling

**Figure 2:**
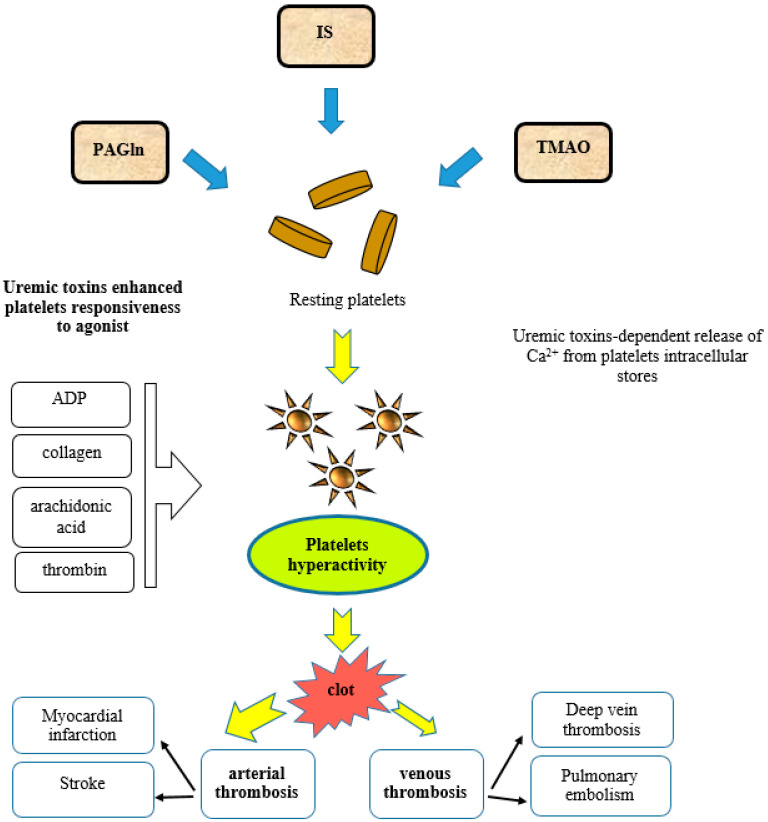
Mechanisms of platelet hyperactivity and platelet-induced thrombosis. IS, indoxyl sulfate; TMAO, trimethylamine N-oxide; PAGln, trimethylamine N-oxide; ADP, adenosine diphosphate.

The plasma metabolite PAGln promotes platelet thrombotic potential by the modulation of platelet responsiveness mediated through G protein-coupled receptors (GPCRs), including alpha 2A, alpha 2B, and beta2-adrenergic receptors (ARs) [107,109]. ARs are expressed on platelets and their activation is connected with platelet stimulus-dependent Ca^2+^ release and responsiveness [110]. It is well known that the activation of ARs is involved in the progression of cardiovascular diseases and has an influence on the platelet function. However, AR signaling involvement in the stimulation of thrombotic complications induced by gut microbiota-derived metabolites such as PAGln is a relatively new discovery [107]. PAGln and PAGly enhance platelet function via stimulus-dependent responsiveness to multiple agonists and the release of intracellular calcium (Figure 2). In arterial injury models, PAGln enhanced the rate of thrombus formation and increased the thrombosis potential [107]. In a murine model of arterial injury, PAGln-induced pro-thrombotic effects were reversed by beta-blocker treatment. Undoubtedly, numerous clinical trials with the use of beta-blockers proved that beta-blocker therapy is highly beneficial in patients with CVD and reduces the risks of heart attacks, strokes, heart failure, and death [111]. Carvedilol, as a non-selective beta-adrenergic receptor blocker (β1, β2) and an alpha-adrenergic receptor blocker (α1), was shown to promote the inhibition of the platelet function [112,113]. This observation can be partially explained by the attenuation of PAGln-triggered AR signaling events.

## 7. Choline/Phosphatidylcholine-Derived Uremic Toxins

### 7.1. Trimethylamine N-Oxide

Trimethylamine *N*-oxide (TMAO) is an organic compound biosynthesized in the liver from trimethylamine (TMA). TMA is generated as a waste product by gut microbiota from food sources rich in quaternary amines such as choline, phosphatidylcholine (lecithin), or L-carnitine when the transport capacity of these substances is exceeded in the small intestine [114]. TMA is absorbed from the intestinal tract and carried via the portal circulation to the liver, where it is subsequently converted by a family of hepatic flavin-containing monooxygenases (particularly FMO3) into TMAO. The high content of specific TMA-containing dietary nutrients, such as choline, lecithin, and L-carnitine, is common in the Western diet pattern characterized by an increased intake of red meat, eggs, and fat-rich products [115]. Choline is an essential nutrient that is used by cells to synthesize membrane phospholipids. Furthermore, choline is the precursor of the neurotransmitter acetylcholine and a major source for methyl groups via its metabolite, trimethylglycine (betaine). The main dietary sources of the choline moiety, which is mostly present in food as lecithin, are eggs, liver, soybeans, and pork. In metabolomics studies, choline was proven to be one of three markers that predicted the development of CKD and remained significant after the adjustment for estimated GFR, age, sex, diabetes mellitus, hypertension, and proteinuria at baseline [116]. Recent literature suggests that the enhanced abundance of choline utilization genes in the intestinal microbiome is associated with increased TMA levels in the gut and, subsequently, with a higher hepatic production of TMAO. Also, the consumption of a high-fat diet has been reported to elevate circulating TMAO levels in humans. TMAO is a predominant kidney-cleared metabolite, excreted in the urine in part through tubular cell secretion. Organic cation transporters (OCT1 and OCT2) have a crucial role in the urinary excretion of TMAO in mice, however, the role of OCT2-mediated tubular secretion is questioned in humans [117]. Also, transporters of the ATP-binding cassette family, including ABCG2 (BCRP) and ABCB1 (MDR1) are involved in this process [117]. Reduced renal clearance results in the accumulation of TMAO and circulating TMAO levels are elevated in patients with reduced GFR. Plasma TMAO was higher among CKD grade 3–5 patients than in people with proper kidney function [118]. Due to the fact that TMAO is not a protein-bound toxin, it is removed by hemodialysis. Also, the plasma levels of TMAO are significantly reduced following renal transplantation [119]. Additionally, TMAO can also be excreted in sweat and exhaled air. The physiological role of TMAO in humans is unknown. The relationship between CKD and an increased level of TMAO was described in the early 90 s [120]. Since then, multiple clinical studies have demonstrated that circulating higher TMAO levels are associated with systemic inflammation, an increased cardiovascular risk, and adverse CVD events such as heart attacks, strokes, and death [121]. According to the Framingham Heart Study and the analysis of plasma from 1434 involving participants with normal renal function at baseline, it was reported that elevated choline and TMAO levels are associated with a risk of future CKD development. High levels of TMAO contribute to the 2.8-fold increased risk of mortality in CKD patients [122]. Numerous studies confirmed that microbiota-derived TMAO generation shows a dose-dependent association with the risk of arterial thrombosis. In mice, a decreased time to occlusion of the carotid artery was shown after an intraperitoneal injection of TMAO [123]. Also, in numerous clinical cohorts, an association of high TMAO levels with an increased risk of thrombotic events, such as a heart attack or stroke, was observed [124]. Moreover, the association between TMAO and thrombosis incidents’ risk was observed even following adjustments for CVD history, traditional CVD risk factors, renal function, and medication use. The mechanisms by which TMAO fosters enhanced thrombotic risks include the modulation of platelet function, increased TF expression, and vascular inflammation.

#### 7.1.1. Platelet Hyperactivity

The modulation of platelet function and the generation of a pro-thrombotic phenotype by TMAO confirm the role of gut microbes in thrombosis (Figure 2). In human studies, healthy volunteers that were orally supplemented with choline showed an increased TMAO circulating level, enhanced platelet aggregation, and responsiveness to agonist [123,125]. Even in participants on low-dose aspirin, higher TMAO levels were dose-dependently associated with increased platelets’ aggregation responsiveness [126]. In experimental models, under shear stress conditions, TMAO generated platelet hyperactivity [123]. Mice receiving TMAO showed an enhanced prothrombotic phenotype and increased platelet responsiveness. TMAO-dependent enhancement in Ca^2+^ release from platelet intracellular stores heightened their responsiveness to submaximal agonist stimulation such as thrombin, ADP, arachidonic acid, and collagen [123]. The stimulation of platelets with TMAO resulted in Ca^2+^ release which was induced by the inositol 1,4,5-trisphosphate (IP_3_) signaling pathway. When IP_3_ binds to its receptor, calcium is released into the cytosol and activates various calcium-regulated intracellular signals [123].

The animal studies showed that specific inhibition of the microbial choline TMA-lyase, which is involved in the production of TMA from choline, leads to a significant reduction in plasma TMAO levels, a recovery from dietary-induced platelet aggregation and thrombus formation [127].

#### 7.1.2. Endothelial Cells

Vascular inflammation is involved in the pathogenesis of thrombotic complications. TMAO induces vascular inflammation which manifests as an increased expression of endothelial extracellular matrix, the production of proinflammatory cytokines, and adhesion molecules [128]. Vascular inflammation was triggered after an acute infusion of TMAO via enhanced proinflammatory gene expression induced by mitogen-activated protein kinase (MAPK) and NF-κB nuclear translocation signaling pathway [129]. Mouse aortas, after an acute injection of TMAO, showed an increased expression of vascular adhesion molecules, such as E selectin and intercellular cell adhesion molecule-1 (ICAM-1). TMAO also increased endothelial cell oxidative stress and the expression of vascular cell adhesion molecule-1 (VCAM-1) [130,131]. The inflammation in endothelial cells and the arterial vascular wall in mice was induced by NOD-, LRR-, and pyrin domain-containing protein 3 (NLRP3) inflammasome formation and activation [132,133,134,135]. The exact mechanisms by which TMAO induces inflammasome activity are not exactly known.

#### 7.1.3. Tissue Factor

Recent studies have shown that beyond impacting platelet function, TMAO induces expression of TF by activation of the NF-κB signaling pathway (Figure 1) [136]. Vascular TF promotes thrombosis and vascular inflammation. The stimulation of human umbilical vein endothelial cells (HUVECs) with TMAO shows a dose-dependent increase in mRNA for TF [137]. Also, mice treated with TMAO significantly increased IL-1β production in the intima, which is a potent inducer of TF expression [132].

## 8. Thrombosis Prevention

A promising therapeutic strategy to reduce the risk of thrombosis in CKD patients is to target the production of uremic toxins by the gut microbiota. This can be achieved by altering the microbial function and/or composition of the intestinal flora as well as by introducing dietary manipulation. In CKD patients, the widespread use of various types of drugs or dietary supplements such as potassium binding-resins may affect the production of metabolites by the gut-microbial community. Experimental and clinical studies were conducted to assess the role of prebiotics, probiotics, and fecal microbiome transplants in the regulation of microbial function and composition. Also, direct inhibition of microbial enzymes was considered as a potential therapeutic approach for preventing or reducing adverse health outcomes. However, little is known about the influence of these interventions on the production of uremic toxins and the reduction of the thrombotic risk in CKD patients. Recently, it was shown that targeted inhibition of gut microbial TMAO production reduces the thrombosis potential in mice [138]. The suppression of TMAO levels with choline TMA lyase inhibitors significantly reduced platelet aggregation and adherence to collagen, without enhancement of bleeding risk. These halomethylcholine-based inhibitors lead to irreversible inactivation of choline conversion into TMA by choline TMA lyase enzyme. Specifically, these inhibitors act on microbial catalytic choline utilization protein C (CutC) and its activating partner—choline utilization protein D (CutD). The reduction of TMA formation results in the suppression of TMAO levels that leads to a decreased platelet responsiveness and a thrombotic potential.

Another way to attenuate uremic toxin production is to modify the gut environment. Using only probiotics, which are live microorganisms that provide health benefits by improving and restoring gut microflora, was ineffective in attenuating uremic toxicity and resulted in adverse clinical outcomes [139,140]. This is most likely due to the fact that the uremic milieu affects the gut environment. In such cases, it is impossible to restore a normal microbiome by providing only fresh microorganisms. Therefore, prebiotic administration seems to be an interesting therapeutic option in CKD patients. Prebiotics are non-digestible compounds in food that stimulate the growth and activity of beneficial bacteria, such as *Bifidobacteria* and *Lactobacilli* by increasing the availability of carbohydrates to be fermented by the microbiota. Preliminary experimental and clinical studies examining the impact of prebiotics on gut-derived uremic toxins production were very encouraging [141,142,143]. However, in the randomized controlled trial testing the effect of prebiotic (fructooligosaccharide, FOS) vs. placebo in a group of 50 non-diabetic CKD patients (eGFR < 45 mL/min/1.73 m^2^), there were no differences in the concentration of IS and IAA between groups [144]. Researchers found only a non-significant potential of FOS in reducing serum total and free PCS. None of these studies tested the hypothesis concerning possible thrombotic risk reduction in examined groups.

The promising effect in uremic toxins reduction can be achieved by implementing a synbiotic strategy. This strategy involves a combined use of prebiotics plus probiotics. In a randomized controlled trial, after the implementation of synbiotic therapy, a reduction of PCS concentration but not IS was shown [145]. However, similarly to previously-described studies, the scientists did not assess any possible effects of these interventions on the thrombotic potential.

Recently, the association between physical activity and gut microbiota composition has been the item of many studies [146]. Intestinal microbiome modulation is related to physical exercises. Physical effort stimulates a high noradrenaline plasma level that impacts the growth of nonpathogenic commensal bacteria [147]. Additionally, the increase in vagal tone may decrease intestinal permeability [148]. Also, physical activity improves immunity by decreasing pro-inflammatory cytokine production. Finally, physical activity improves intestinal peristalsis, which decreases the contact of uremic toxins with the gastrointestinal mucus layer. In fact, the direct relationship between physical exercise and modulation of gut microbiota in ESRD has not been studied and this association is poorly supported by the scientific evidence among other CKD groups. However, this strategy seems to be promising in improving patient outcomes [149,150]. Therefore, physical exercise in CKD patients may represent a novel nonpharmacological approach to modulate gut microbial composition.

## 9. Conclusions

**Figure 3:**
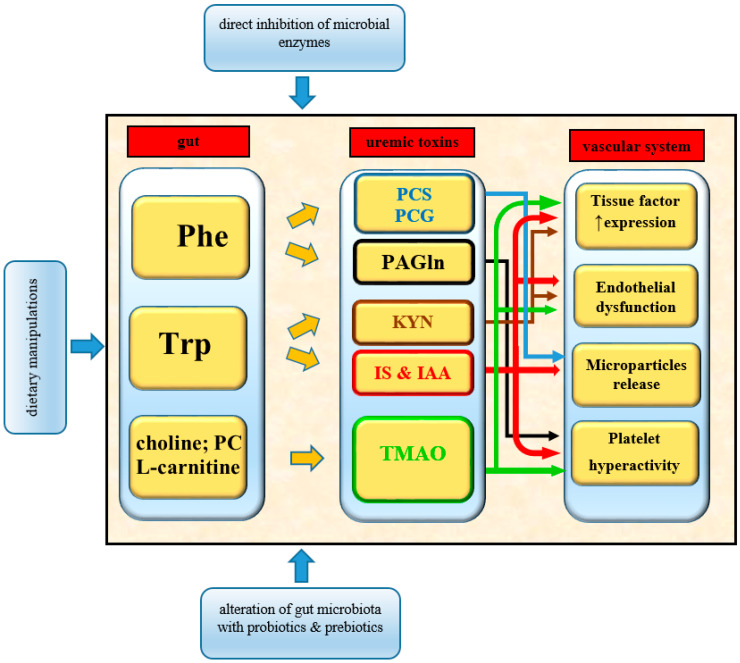
Mechanisms of thrombolome generation and action in CKD. CKD, chronic kidney disease; Phe, phenylalanine; Trp, tryptophan; PC, phosphatidylcholines; PCS, p-cresol sulfate; PCG, p-cresol glucuronide; PAGln, phenylacetylglutamine; IS, indoxyl sulfate; IAA, indole-3-acetic acid; KYN, kynurenine; TMAO, trimethylamine N-oxide.

During the past decade, experimental and clinical studies revealed that gut microbiota-generated uremic toxins play a critical role in cardiovascular complications such as arterial and venous thrombotic events. It was determined that prothrombotic phenotype induced by uremic toxins (TMAO, IS, IAA, PAGln, PCS, and PCG) is associated with critical mechanisms such as platelet hyperactivity, endothelial dysfunction, microparticles release, and an increased expression of tissue factor (Figure 3). The following attempts were made to decrease gut microbiota-derived uremic solutes: pre- or probiotic modulation of commensal microflora, direct inhibition of bacterial enzymes involved in uremic toxin production, and reducing the amount of supplied nutrients containing their precursors [144,145]. These discoveries provide a new potential therapeutic target to prevent thromboembolic complications in CKD patients. Nowadays, it is evident that gut flora is an incredibly diverse community. Different CKD stages, including pharmacological and non-pharmacological treatment, may alter the intestinal microbiota composition and their properties. This research area offers many exciting opportunities to discover new metabolites and pathogenic pathways influencing the development of thrombotic complications among CKD patients. These findings may also help develop a better understanding of thrombotic mechanisms in other diseases.

## Figures and Tables

**Table 1:** Microbial families that are more and less abundant in ESRD containing phosphotransbutyrylase, butyrate kinase, urease, tryptophanase, and p-cresol producing enzymes [42].

	Gut Microbiota		Enzyme Possessed by Bacteria
	Phylum	Family	
More abundant in ESRD	Actinobacteria	Cellulomonadaceae	urease
		Dermabacteraceae	urease
		Microccaceae	urease
	Firmicutes	Clostridiaceae	urease; tryptophanase; p-cresol production enzymes
	Proteobacteria	Polyangiaceae	urease
		Alteromonadaceae	urease
		Enterobacteriaceae	urease; tryptophanase; p-cresol production enzymes
		Methylococcaceae	urease
		Halomonadaceae	urease
		Moraxellaceae	urease
		Pseudomonadaceae	urease
		Xanthomonadaceae	urease
	Verrucomicrobia	Verrucomicrobiaceae	urease; tryptophanase
Less abundant in ESRD	Bacteroidetes	Prevotellaceae	phosphotransbutyrylase; butyrate kinase
	Firmicutes	Lactobacillaceae	Phosphotransbutyrylase; butyrate kinase
	Proteobacteria	Alcaligenaceae	-

## Data Availability

This is a review paper and thus the data presented in this study are openly available in published papers listed in References.
